# The Impact of the Cancer Microenvironment on Macrophage Phenotypes

**DOI:** 10.3389/fimmu.2020.01308

**Published:** 2020-06-23

**Authors:** Sunyoung Ham, Luize G. Lima, Erica Lek, Andreas Möller

**Affiliations:** ^1^Tumor Microenvironment Laboratory, QIMR Berghofer Medical Research Institute, Herston, QLD, Australia; ^2^Faculty of Health, School of Biomedical Sciences, Queensland University of Technology, Brisbane, QLD, Australia

**Keywords:** tumor microenvironment, tissue-resident macrophage, monocyte-derived macrophage, small extracellular vesicles, tumor-derived cytokines

## Abstract

Within the tumor microenvironment, there is an intricate communication happening between tumor and stromal cells. This information exchange, in the form of cytokines, growth factors, extracellular vesicles, danger molecules, cell debris, and other factors, is capable of modulating the function of immune cells. The triggering of specific responses, including phenotypic alterations, can ultimately result in either immune surveillance or tumor cell survival. Macrophages are a well-studied cell lineage illustrating the different cellular phenotypes possible, depending on the tumor microenvironmental context. While our understanding of macrophage responses is well documented *in vitro*, surprisingly, little work has been done to confirm these observations in the cancer microenvironment. In fact, there are examples of opposing reactions of macrophages to cytokines in cell culture and *in vivo* tumor settings. Additionally, it seems that different macrophage lineages, for example tissue-resident and monocyte-derived macrophages, respond differently to cytokines and other cancer-derived signals. In this review article, we will describe and discuss the diverging reports on how cancer cells influence monocyte-derived and tissue-resident macrophage traits *in vivo*.

## Introduction

Macrophages are key immune cells involved in the phagocytosis of foreign factors and debris, and the production of cytokines (1–3). Classic macrophages can respond to cancer cells upon exposure to tumor-associated antigens (4). However, macrophages that are associated with established tumors are usually known to produce anti-inflammatory cytokines and support tumor progression (5). These cancer-associated types of macrophages have also been associated with metastasis, including early metastatic steps such as pre-metastatic niche formation (6).

Macrophages were first described by Elie Metchnikoff in 1882, as mononuclear phagocytic cells important for animals' defense against bacterial infection (1). In the context of cancer, initial studies focused on their phagocytic function, and how their secretory products could act as proinflammatory and anti-tumor agents (1, 7, 8). For example, breast cancer patients presenting with tumor masses highly infiltrated by macrophages had a reduced risk of metastasis (9). In contrast, during 1990s, proangiogenic and protumoral roles for macrophages started to be suggested in diverse cancer types, including breast (10), lung (11), and ovarian cancer (12).

Studies on the biology of monocyte differentiation to macrophage helped understand the opposing inflammatory and anti-inflammatory roles of macrophages (13, 14). These studies demonstrated how cytokines are involved in monocyte differentiation. For example, Interferon-gamma (IFN-γ) assists in the initiation of immune responses (15). Macrophage activation by lipopolysaccharide (LPS) results in an increase in IL-12 production, a cytokine commonly found in the inflammatory environment (16). When macrophages are treated with LPS in combination with IFN-γ, for example, IL-12 secretion is up-regulated, with subsequent promotion of Th1 inflammatory responses (17). On the other hand, macrophages exposed to IL-4 or IL-10 are known to promote Th2 anti-inflammatory responses (18). Charles Milles introduced that macrophages activated by LPS and IFN-γ are commonly termed M1 macrophages; while IL-4 or IL-10 treated macrophages are termed M2 macrophages (19). Nevertheless, this simple M1/M2 macrophage polarization, induced by only several cytokines, is not sufficient to describe the broad macrophage variability observed in different disease models (20), particularly in cancer (21).

So far, most work has been done on investigating macrophage responses to recombinant cytokines in *in vitro* cell culture settings. Mostly, murine and human macrophage cell lines, including RAW and THP-1 cells, respectively, have been used for those studies. Based on this data, we have generated a thorough understanding of signaling pathways in macrophages, in response to cytokines and other stimuli. Intriguingly, there is a paucity of studies on how macrophages in *in vivo* cancer microenvironments respond to cytokines. In fact, some reports highlight stark discrepancies between the responses of cell-cultured macrophages to a cue when compared to the macrophages in a tissue context (22). For example, comparison of bone marrow-derived macrophage (BMDM) and Raw 264.7 cells by RNA sequencing and proteomics uncovered dissimilarity in response to inflammation (23, 24). Additionally, there are at least two distinct macrophage populations, with different origins and functions, present in a tumor. Our knowledge of the different roles these populations have to play in different phases of tumor progression and metastasis are even more limited. We will now discuss the state of knowledge for these macrophage populations in *in situ, in vivo* or *ex vivo* cancer microenvironmental settings.

## Monocyte-Derived Macrophages and Tissue-Resident Macrophages: Origins and Phenotypes

Based on their origin, macrophages are classified into monocyte-derived macrophages or tissue-resident macrophages (25). Monocyte-derived macrophages originate from adult hematopoietic stem cells in the bone marrow (26). These macrophages are firstly distributed to tissues as monocytes, which can then differentiate to macrophages depending on organ-specific cues and circumstance (27). On the other hand, tissue-resident macrophages are suggested to originate from progenitor cells during embryonic or fetal development, and are not dependent on adult hematopoiesis (28, 29). These macrophages have self-renewal properties, as well as distinct features and names that depend on the organ in which they reside (30). The tissue-resident macrophages' nomenclature includes historical names, such as bone marrow, microglia (brain) (31), alveolar (lung) (32), Kupffer (liver) (30), and kidney macrophage (33).

**Figure 1:**
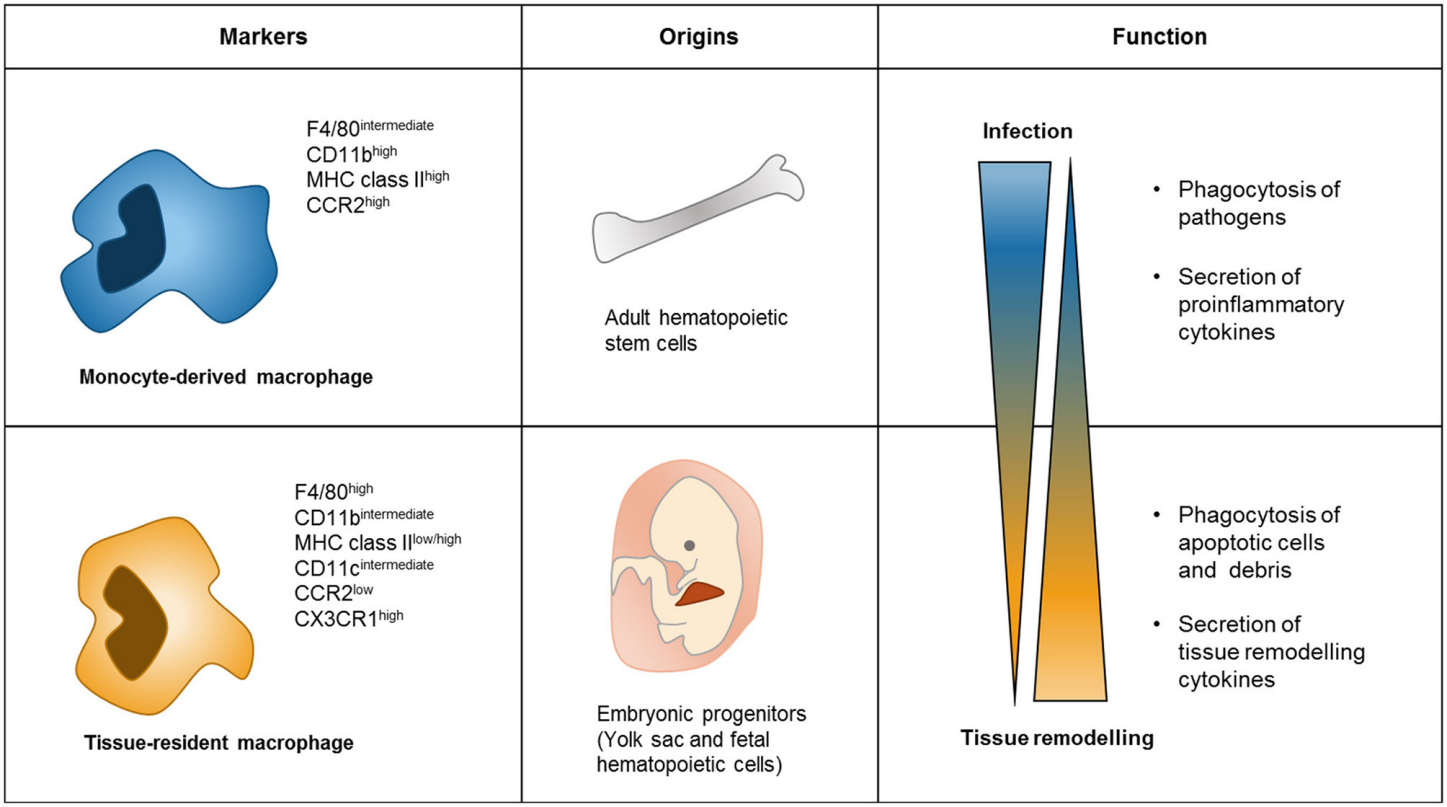
Difference between Monocyte-derived macrophages and tissue-resident macrophages. Monocyte-derived macrophages commonly express high levels of CD11b MHC class II and CCR2, while tissue-resident macrophages have high levels of F4/80 and CX3CR1 (30). Monocyte-derived macrophages begin from adult hematopoietic stem cells through monocyte differentiation. However, progenitors of tissue-resident macrophages are of embryonic origin and fetal hematopoietic cells, and maintain their number by self-renewal signaling in tissue (30). Functions of these two types of macrophages are different as well. Monocyte-derived macrophages act in infection conditions to phagocytosis pathogens and secretes cytokines related to proinflammatory conditions (35). In contrast, tissue-resident macrophages subsist in tissue for maintaining tissue homeostasis by phagocytosis apoptotic cells and secreting cytokines related to tissue remodeling (3).

Tissue-resident macrophages are highly heterogeneous, showing more variable levels of transcription factors and surface markers compared to monocyte-derived macrophages (Figure 1). Regarding the expression of surface markers, monocyte-derived macrophages are generally F4/80^intermediate^/CD11b^high^/MHC class II^high^/CCR2^high^, while tissue-resident macrophages are usually identified by the F4/80^high^/CD11b^low^/Cx3CR1^high^/MHC class II^high/low^/CCR2^low^ immunophenotype (30). In addition, it has been suggested that the responses triggered within the cancer microenvironment are different between monocyte-derived macrophage and tissue-derived macrophages. In pancreatic tumor, for example, tissue-resident macrophages proliferate, and promote tumor progression and pro-fibrotic activity, while monocyte-derived macrophages do not affect tumor progression, but have potent roles in antigen presentation (34). Conversely, monocyte-derived macrophages accumulate in high numbers during lung injury, whereas tissue-resident macrophages persist in their numbers (35). Moreover, *in vivo* injections of either LPS or IL-4 trigger different responses in monocyte-derived and tissue-derived macrophages, both functionally and phenotypically (35, 36). These studies show that monocyte-derived and tissue-resident macrophages can display distinct characteristics in different conditions (Figure 1). Therefore, it is important to clearly identify these two populations of macrophages when assessing their roles in the tumor microenvironment, particularly how both cell subsets are differentially affected by tumor-derived factors.

## Macrophages in the Cancer Microenvironment

The surrounding environment of macrophages has been reported to determine their polarization (37). For example, macrophages in either hypoxic or acidic conditions promote tumor progression more efficiently than cells derived from normoxic condition (38–40). This observation indicates that macrophage differentiation can be altered by extrinsic factors, hence impacting the cancer microenvironment. There is a vast difference between the cytokine profiles secreted from normal and cancer cells. It is known that many types of cancer cells secret a large range of extracellular mediators, including cytokines, chemokines and growth factors, such as the chemokine (C-C motif) ligand 2 (CCL2), IL-6, transforming growth factor beta (TGF-β), tumor necrosis factor alpha (TNF-α), matrix metalloproteinases (MMPs), and granulocyte-macrophage colony-stimulating factor (GM-CSF) (41, 42). These proteins play important roles in altering the phenotype of macrophages, but might affect monocyte-derived macrophages and tissue-resident macrophages differentially. The composition and amounts of cytokines secreted within the tumor microenvironment by both cancer and stromal cells partially determine the reaction of macrophages to a cancer, which could result in either promotion or suppression of immune responses, and consequent inhibited or sustained cancer growth, respectively.

## Effects of Tumor-Derived Cytokines on Monocyte-Derived Macrophages

CCL2 is known to be highly abundant in the cancer microenvironment (43). It is shown to recruit CCR2^+^ highly inflammatory monocytes, which are then differentiated to F4/80^+^/CD11b^+^/Gr1^−^ macrophages, promoting metastasis through VEGF-dependent mechanisms in breast cancer (44). Furthermore, F4/80^+^/CD11b^+^macrophages express higher levels of CCR2 than tissue resident macrophages, being recruited by CCL2 secreting tissues. CCL2, in turn, regulates CCR2^+^ macrophage signaling and induces, for example, secretion of CCL3 and consequent extravasation of cancer cells (45). Deletion of CCL3 in bone marrow-derived macrophages suppresses lung metastasis and reduces the recruitment of monocyte-derived macrophages to tumor site (45). In another study, CCL2 has been reported to play an important role in differentiating monocytes. CCL2 was incubated with human CD11b^+^ monocytes, causing them to differentiate to a CD14^+^/CD206^+^/CD11b^+^ population (46). These macrophages produce protumoral cytokines and are associated with a tumor promoting phenotype (46).

Several publications have demonstrated that IL-6 is also associated with macrophage polarization. For example, in the glioblastoma microenvironment, IL-6 and CSF-1, which are produced by glioblastoma-associated endothelial cells, increase arginase-1 expression, which is mediated by HIF-2α activation in monocyte-derived macrophages (47). Therefore, when IL-6 is knocked out in glioblastoma-associated endothelial cells, glioblastoma-bearing mice display an increased survival rate (47). Moreover, macrophages generated from peripheral blood monocytes and incubated with macrophage colony-stimulating factor (M-CSF) induce high levels of DC-SIGN, which could also be achieved by incubation of monocytes with conditioned media from cancer cell lines containing high levels of IL-6 and IL-10 (48). The DC-SIGN protein expression is commonly observed in macrophages found in patient tumor stroma, and has been reported to be associated with high levels of VEGF, as well as a proangiogenic phenotype (48, 49). TGF-β is also known to be secreted by various cancer cells, including breast (50), lung (51), and liver cancer cells (52). Among the cytokines secreted from hepatocellular carcinoma, TGF-β induced Tim3 signal, activated NF-kB and STAT6, increased IL-6 and IL-10, and decreased IL-1 in macrophages (53). The IL-6 released from these macrophages, but not IL-10 and GM-CSF, suggests that cancer cells are promoting their proliferation in a paracrine manner (53). When TGF-β is blocked by antibodies, human monocyte-derived macrophages differentiated by M-CSF have increased secretion of IL-10 and decreased levels of IL-12 (54). High levels of TNF-α are found in cancer and non-cancer proinflammatory environments (55). TNF-α produced by the cancer affects macrophage SINGLEC1 expression, which is found in high levels on tumor-associated macrophages (56). SINGLEC1 and CCL8 expression on macrophages are independent prognostic markers for poor survival (56). Taken together, these observations show that monocyte-derived macrophages secrete cytokines associated with tumor promotion or contribute to the cancer progression in response to cancer-derived cytokines.

## Effect of Tumor-Derived Cytokines on Tissue-Resident Macrophages

There has been less research done on the role and responses of tissue-resident macrophages in the tumor microenvironment, leaving us with a sketchier understanding. However, several studies show distinguishing features of monocyte-derived and tissue-resident macrophages in tissues (2, 57). Additionally, there are several reports showing that tissue-resident macrophages are involved in tissue remodeling rather than inflammatory conditions (58). Despite these reports, it is still not clear what the exact role(s) of this tissue-resident macrophage in the tumor microenvironment are. It has been reported that the recruitment of macrophages in CCR2^−/−^ mice to pancreatic ductal adenocarcinoma tumors were reduced (34). However, it was noted that this reduction did not affect pancreatic ductal adenocarcinoma tumor growth (2). This result suggests that pancreatic cancer growth is regulated by CCR2^+^ monocyte/macrophage-independent mechanisms (2). In contrast, this report demonstrated that these tissue-resident macrophages are able to self-renew in tumors due to tumor-derived CSF-1 and promote tumor progression (2). A recent study partially explained this phenotype by showing that CSF1/2 regulates both proliferation and angiogenic capacity of cardiac tissue-resident macrophage by regulating KFL4 levels (59). In ovarian cancer, bioinformatics analyses showed that tumor-associated macrophages are similar to tissue-resident macrophages, but not monocyte macrophages (60). At the same time, the phenotype and function of tumor-associated macrophages accumulating in breast cancer tumors is different from that of breast tissue-resident macrophages (61). As such, macrophage characteristics in each organ are different, and it differs in distinct tumor microenvironments. How the different macrophage types respond in a given situation in a cancer would therefore require further detailed studies.

## Tumor-Derived Extracellular Vesicles

Small extracellular vesicles (sEVs) are derived from cells without being able to self-replicate (62). Fusion of multi-vesicular bodies (MVBs) with the plasma membrane allows the release of sEVs into the extracellular environment (63). These sEVs play an important role in the intercellular communication within the tumor microenvironment. Depending on the origin of the cell, the extracellular vesicles have a different content, and therefore, the extracellular vesicles released from the cancer cells are different from the corresponding normal cells (64). sEVs contain various bioactive compounds such as proteins, lipids, mRNAs and microRNAs (65–67). It has been reported that tumor-derived sEVs can be distributed to various organs and lymph nodes through blood and/or lymphatic vessels (68). These vesicles are not only found to be retained in distal tissues, but have also shown to be taken up, for example, by cells in the brain and bone marrow (68, 69). Therefore, it is reasonable to suggest that tumor-derived EVs are capable of changing the characteristics and behavior of their target cells. As such, these compounds have been shown to affect the phenotype and cellular function of recipient cells in different organs, including immune cells, and especially macrophages.

## Effects Tumor-Derived Extracellular Vesicles in Monocyte-Derived Macrophages

Breast cancer-derived sEVs containing high levels of gp130 activate STAT3 signaling in monocyte-derived macrophages (70). The proportion of CD163^+^CD206^high^HLA-DR^low^ macrophages, derived from human blood CD14^+^ monocytes, was shown to be increased after incubation with hypoxic lung cancer-derived extracellular vesicles enriched for microRNA-103a (71). MicroRNA-103a in turn targets PTEN to activate AKT and STAT3 pathways (71). Y RNA, hY4 is another small non-coding RNA found in sEVs from chronic lymphocytic leukemia (72). These sEVs induced secretion of CCL2, CCL4, and IL-6, as well as expression of PD-L1, in monocytes and macrophages (72). Furthermore, these macrophages with activated AKT and STAT3 signaling pathways, and increased levels of PD-L1 induce a reduced immune response and therapeutic resistance (73). Altogether, these studies suggest that sEVs derived from cancer cells are capable of polarizing monocyte-derived macrophages to tumor-associated macrophages.

## Effects of Tumor-Derived Extracellular Vesicles on Tissue-Resident Macrophages

Recently, there have been a number of reports suggesting that tumor-derived EVs induce premetastatic niche formation by altering myeloid cell phenotypes, including that of monocytes and macrophages (74, 75). Apart from these publications, there has not been much research reported on the impact of tumor-derived sEVs on tissue-resident macrophages. A few examples on the role of sEVs come from glioblastoma and microglia work, possibly due to brain-resident macrophages being mostly of embryonic origin (30). Glioblastoma stem-like cells secrete EVs, which induce membrane type 1-MMP (MT1-MMP) in microglia with strong tumor-supportive functions (76). Similarly, these vesicles are enriched for miR-451 and miR-21, which are transferred to microglia. In these cells, the microRNAs decrease c-myc levels, inducing gene expression alterations in favor of protumoral phenotypes (77). Additionally, sEVs might change extracellular matrix composition via MT1-MMP and c-myc, which induces cancer cells migration and invasion (78, 79). However, more research is required to understand the interactions and roles cancer-secreted EVs exert on tissue-resident macrophages.

## Conclusion

**Figure 2:**
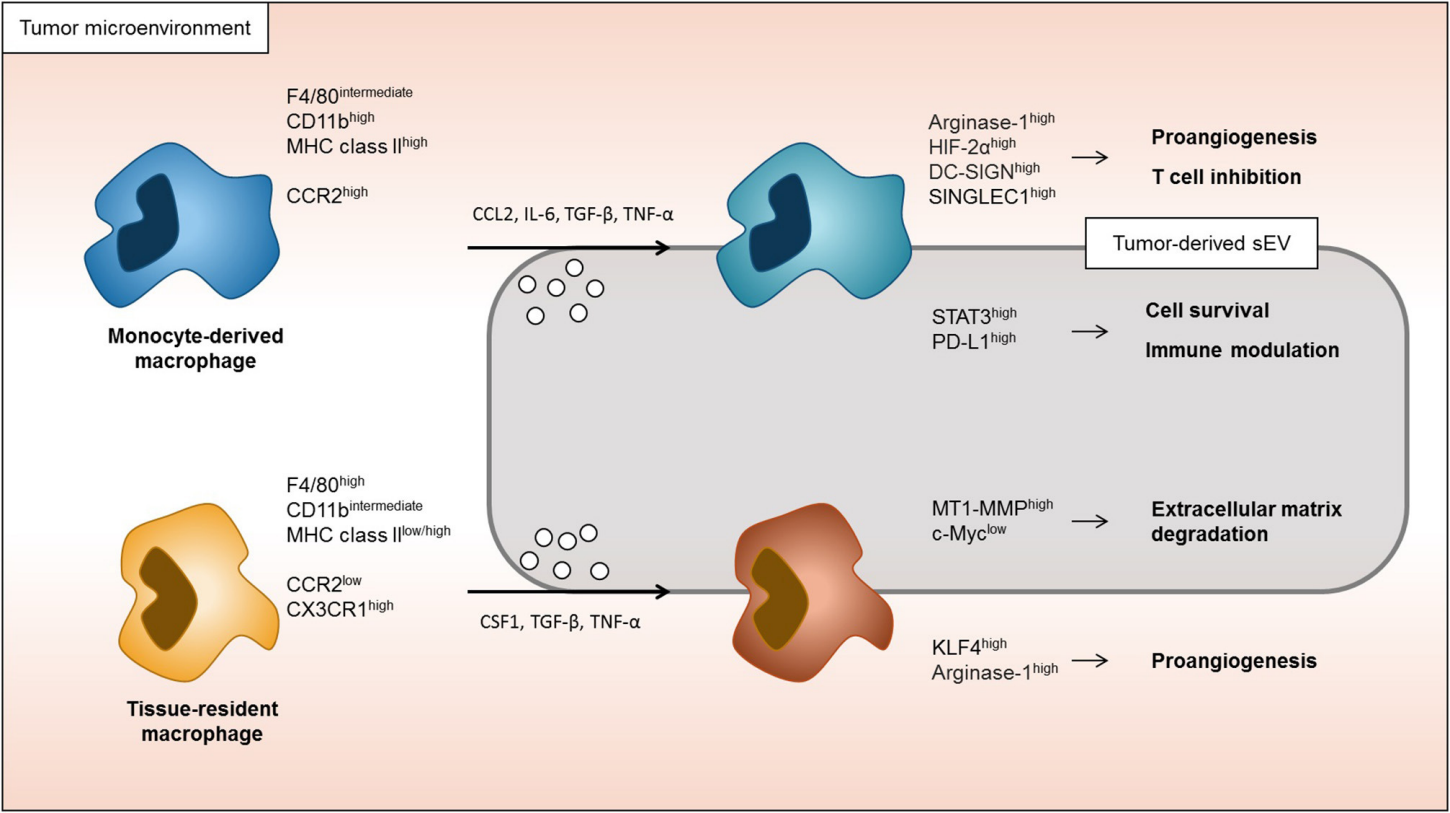
Response of monocyte-derived macrophages and tissue-resident macrophages in tumor microenvironment. Both monocyte-derived macrophages and tissue-resident macrophages are able to be associated to tumor progression. Monocyte-derived macrophages in tumor microenvironment are more induced to immune modulation and macrophage cells survival signaling. On the other hand, tissue-resident macrophages are more associated to the change in extracellular matrix and proangiogenic signaling.

Although much research has been conducted on understanding the responses of macrophages to cytokines and EVs, most of these studies were done in cell culture settings. While very informative, more recent literature suggests that the tissue-context dimension, which cannot be mimicked in cell culture, has an enormous impact on macrophage responses. Without a detailed knowledge of the macrophage-lineage specific response to certain stimuli in the tumor microenvironment context, it is very difficult to ascertain the roles and therefore, the best macrophage-targeting interventions in a cancer setting. This review described how tumors affect monocyte-derived and tissue-derived macrophages by dividing the roles of soluble factors and EVs (Figure 2). This summary and interpretation highlight some areas of cancer macrophage biology requiring further research so we can better understand the intricate relationship between cancer cells and macrophages. Many studies have evaluated the tumor microenvironment by using conditioned media, focusing on the role of single or small sets of cytokines and/or sEVs. However, regional differences within the microenvironment as well as spatial/regional regulation mechanisms of macrophages are not understood at any depth (69). It will be very exciting, using more comprehensive approaches in animal models and patient samples, to interrogate on single cell levels *in situ* how macrophages phenotypes are differentially regulated with important implications for our understanding of cancer progression and developing novel therapeutic approaches. Recently, many studies have been conducted targeting tumor-associated macrophages. For example, it has been reported that activation of CD206 by RP-182 on tumor-associated macrophages reprograms these cells and increases their anti-tumor activity (80). In addition, increased legumain, a lysosomal peptidase, in tumor-associated macrophages increases CD8 T cell activity (81). Understanding the macrophage origin and how these types of macrophages respond to the tumor microenvironment will be essential to develop better cancer therapies in the future.

## Author Contributions

SH and AM wrote and reviewed the manuscript. LL and EL edited and reviewed the manuscript. All authors contributed to the article and approved the submitted version.

## Conflict of Interest

The authors declare that the research was conducted in the absence of any commercial or financial relationships that could be construed as a potential conflict of interest.
